# Renal Presentation in Pediatric Acute Leukemia: Report of 2 Cases: Erratum

**DOI:** 10.1097/01.md.0000472590.81143.a8

**Published:** 2015-10-09

In the article “Renal Presentation in Pediatric Acute Leukemia: Report of 2 Cases”,^1^ which appeared in Volume 94, Issue 37 of *Medicine*, Dr. Naglaa M. Kamal's first name was initially omitted. The article has since been corrected online.
